# Demographic, Clinical and Immunogenetic Profiles of a Greek Cohort of COVID-19 Patients

**DOI:** 10.3390/life11101017

**Published:** 2021-09-27

**Authors:** Maria. G. Detsika, Chara Giatra, Vasiliki Kitsiou, Edison Jahaj, Theofilos Athanassiades, Diamanto Kouniaki, Stylianos E. Orfanos, Ioanna Dimopoulou, Maria Pagoni, Katerina Tarassi, Alexandra Tsirogianni, Anastasia Kotanidou

**Affiliations:** 1 Department of Critical Care Medicine & Pulmonary Services, School of Medicine, G.P. Livanos and M. Simou Laboratories, Evangelismos Hospital, National and Kapodistrian University of Athens, 10676 Athens, Greece; mdetsika@med.uoa.gr (M.G.D.); edison.jahaj@gmail.com (E.J.); stylianosorfanosuoa@gmail.com (S.E.O.); idimo@otenet.gr (I.D.); 2 Hematology Department, BMT Unit, “Evangelismos” Hospital, 10676 Athens, Greece; xgiatra@gmail.com (C.G.); marianpagoni@yahoo.com (M.P.); 3 Immunology-Histocompatibility Department, “Evangelismos” Hospital, 10676 Athens, Greece; vkitsiou@yahoo.gr (V.K.); theofilosathanassiades@yahoo.gr (T.A.); kouniakitzeni@yahoo.gr (D.K.); katerinatarassi@gmail.com (K.T.); alextsir@gmail.com (A.T.)

**Keywords:** SARS-CoV-2, coronavirus disease 2019 (COVID-19), immunology, human leucocyte antigens, immunogenetics

## Abstract

The present cross-sectional study consists of a comprehensive analysis of epidemiological, laboratory, and clinical characteristics of COVID-19 patients in relation to their immunogenetic profiles. We studied 125 COVID-19 patients comprising different stages of disease severity; non-hospitalized (mild *n* = 69) and hospitalized (*n* = 56). Analysis of disease characteristics revealed no major differences between males and females of each group of patients while hospitalized patients were older and presented with comorbidities. A positive allele association was observed for HLA-DRB1*01 in total COVID-19 patients versus healthy controls. Subgrouping of COVID-19 patients in mild and hospitalized further identified a statistically significant increase in HLA-DRB1*01 in mild COVID-19 patients versus controls. The frequency of A*11, A*23, and DRB1*09 alleles was higher, while the frequency of C*12 was lower, in hospitalized patients versus healthy controls albeit with uncorrected statistical significance. The identification of specific allele associations may provide useful future markers for disease susceptibility in order to allow successful clinical management of COVID-19 patients.

## 1. Introduction

Since its outbreak in late December 2019 SARS-CoV-2 pandemic remains one of the most serious health emergencies. Although in most individuals, the disease caused by SARS-CoV-2, named coronavirus disease 2019 (COVID-19), is mild with symptoms mimicking those of the common cold, in some cases, it may lead to severe pneumonia and acute respiratory distress syndrome (ARDS) [1]. In these patients, an overwhelming host immune response against SARS-CoV-2 is observed characterized by an increased production of pro-inflammatory cytokines (cytokine storm) as well as by elevated inflammatory markers such as C-reactive protein (CRP) and ferritin.

Associations between viral infectious diseases and genetic factors including the major histocompatibility complex (MHC), mainly human leukocyte antigens (HLA), have been reported previously. A large international study identified both protective and risk HLA alleles for control of chronic HIV infection [2] while associations of specific HLA alleles were shown for spontaneous elimination of hepatitis C viral infection [3].

Various studies on HLA and COVID-19 have emerged since the beginning of the outbreak on small cohorts from different regions. Initial reports from China and Europe described the association of certain alleles with COVID-19 such as HLA-C*07:29 and B*15:27 the prevalence of which was increased in COVID-19 patients or DRB1*15:01, -DQB1*06:02, and -B*27:07, the prevalence of which was increased in a cohort of Italian patients. Although these studies involved fairly small cohorts, they serve to provide preliminary observations on HLA associations with COVID-19 which may prove of great importance in future strategies for disease management.

The aim of the present study was to analyze the demographic and clinical profiles of COVID-19 patients of different severity stages and investigate their immunogenetic characteristics. Furthermore, it provides data on HLA associations with COVID-19 severity in a Greek cohort of patients.

## 2. Materials and Methods

### 2.1. Patients and Controls

A total of 125 COVID-19 unrelated patients confirmed by a positive SARS-CoV-2 polymerase chain reaction (PCR) test were included. The study patient population consisted of 76 men and 49 women with a median age of 53.3 (range 20–86 years). A group of 316 healthy individuals previously typed in our laboratory was used as controls. Both groups (patients and controls) were of Greek origin. Initial comparisons were performed between COVID-19 patients and age-matched controls. Patients were subsequently divided into two groups according to the following criteria for disease severity; patients with mild symptoms who did not require hospitalization (mild) (*n* = 69) (WHO COVID-19 Ordinal scale score: 1–2) with a median age of 46.0 (range 20–69 years) and patients who developed acute respiratory distress syndrome (ARDS) and required hospitalization (WHO COVID-19 Ordinal scale score: 3–5) or required mechanical ventilation and treatment in the intensive care unit (ICU) (hospitalized) (WHO COVID-19 Ordinal scale score: 6–7) (*n* = 56) with a median age of 62.0 (range 29–86 years) [4]. The study received approval from the Evangelismos Hospital Ethics Committee and written informed consent was obtained from each patient. Collection of demographic and clinical data of hospitalized patients was performed through patient’s medical files whereas required data for patients in the mild group were obtained through appropriate questionnaires. Questionnaires from non-hospitalized patients were not retrieved for seven patients.

### 2.2. HLA Typing

Blood samples were collected from all individuals and typed for all known HLA class I (-A*, -B*, -C*) and class II (-DRB1*, -DQB1*) alleles. Genomic DNA was extracted using the Maxwell 16 purification instrument (Promega, Madison, WI, USA). DNA (30 ng/µL) as used for HLA low-resolution molecular typing by PCR-SSOP and/or PCR-SSP molecular techniques using commercial kits LABType SSO Luminex/One Lambda, AllSet+TM GoldSSP/One Lambda (Thermo Scientific, Waltham, MA, USA) and Olerup SSP kits/CareDx (CareDx Brisbane, CA, USA).

### 2.3. Statistical Analysis

Statistical analysis of demographic and clinical data was performed by contingency tables and application of the Fisher’s test or by comparisons by application of non-parametric Mann–Whitney test where appropriate. Analysis was performed using GraphPad Prism version 6.0. Fisher’s exact *p*-value (*p*), Odds Ratio (OR), and 95% Confidence Interval (CI) for HLA typing analysis were calculated using SPSS statistics 17.0 software. Corrected *p* values (pc) were obtained by applying Bonferroni correction. The significance was set at a level of 0.05. Imputation analysis for missing data was not performed.

## 3. Results

### 3.1. Demographic, Clinical and Laboratory Parameters of COVID-19 Patients

A total of 125 COVID-19 patients were recruited and serologically typed. Patients in the mild group were significantly younger (*p* < 0.0001) with a median age of 46 years (range 20–69) compared to hospitalized COVID-19 patients (*n* = 56) with a median age of 62 years (range 29–86). Furthermore, no comorbidities were reported by non-hospitalized patients. To assess whether mild disease might be manifested differently between men and women within the mild group of patients, a detailed collection and analysis of symptomatology was performed. As shown in Table 1 a wide range of symptoms were reported by non-hospitalized patients all of which were within the reported array of COVID-19 symptoms.

Prevalence of symptoms reported was similar in men and women in the mild group with no statistically significant difference for any of the symptoms apart from severe headache which was more frequent in men (*p* = 0.027) and loss of taste which was more prevalent in women (*p* = 0.027). Duration of temperature was also similar between men and women revealing a homogeneous population of patients. In the hospitalized patient population, a higher number of men was observed possibly reflecting the increased severity of COVID-19 in men. As shown in Table 2, there was no difference in median age between men and women. Although comorbidities were present in hospitalized patients, there were no significant differences between men and women of the specific patient group. Similar demographic and clinical characteristics were observed with no significant differences in any of the symptoms reported or the days of sickness prior to admission to a hospital or ICU.

Comparisons of the clinical examination findings and laboratory parameters at admission included X-ray and CT findings which were similar in both women and men as well as PO_2_/FIO_2_ levels (Table 3).

A significant difference in creatinine levels was observed with higher creatinine levels in men compared to women (*p* = 0.018) whereas all other clinical parameters at admission remained similar between hospitalized men and women. A difference in both duration of hospital/ICU stay (days) and outcome (survival) was observed between women and men with a shorter hospital/ICU stay for women and a higher mortality for men albeit it did not reach statistical significance. This could probably be explained by the relatively small number of women in the specific group.

### 3.2. Analysis of Immunogenetic Profiles of COVID-19 Patients

Comparisons of HLA allele frequencies between COVID-19 patients (*n* = 125) and healthy controls revealed two statistically significant positive allele associations with the disease (Table 4). Specifically, the frequency of HLA-DRB1*01 was significantly increased in COVID-19 patients (*p* = 0.002, OR = 2.693, CI = 1.426–5.087) and the significance was maintained after applying Bonferroni correction (pc = 0.03). HLA-DRB1*09 was also found significantly increased in COVID-19 patients (*p* = 0.024, OR = 10.413, CI = 1.152–94.103) but the significance was lost after applying Bonferroni correction (pc = 0.36, NS).

Further analysis of the two subgroups of patients (non-hospitalized and hospitalized) revealed distinct patterns of allele frequency distributions (Table 5) even though these were not statistically significant after Bonferroni correction for HLA alleles. An increased frequency of HLA-A*11 was observed in hospitalized patients compared to controls (*p* = 0.023, OR = 2.3, CI = 1.154–4.585) as well as compared to patients with mild symptoms (*p* = 0.032, OR = 2.952, CI = 1.099–7.931) and also of HLA-A*23 allele between hospitalized patients and healthy controls (*p* = 0.017, OR = 3.125, CI = 1.268–7.700). An increase in the frequency of HLA-B*40:02 (B61) was found only in non-hospitalized patients (*p* = 0.034, OR = 3.057, CI = 1.215–7.691), whereas for hospitalized patients it remained at similar levels compared to healthy controls. The frequency of HLA-C*04 allele was decreased in mild patients in comparison to hospitalized patients (*p* = 0.013, OR = 2.929, CI = 1.258–6.820). Finally, a decrease in HLA-C*12 allele frequency was observed in hospitalized versus non-hospitalized (*p* = 0.039, OR = 0.383, CI = 0.160–0.915) as well as versus healthy individuals (*p* = 0.025, OR = 0.4, CI = 0.2010.904). However, all the above differences did not maintain significance after applying Bonferroni correction.

In order to exclude whether the differences shown could be influenced by the increased prevalence of men in the patient population, the frequencies for each allele were also assessed between male and female individuals of each group of patients as well as in healthy volunteers (Supplementary Tables S1–S3, respectively). All alleles showing a significant difference showed a similar frequency in males and females thus excluding the influence of gender in the results of the study. Additionally, an increased frequency was revealed in hospitalized patients versus healthy controls for HLA-DRB1*01 allele (*p* = 0.015, OR = 2.508, CI = 1.226–5.127) and for -DRB1*09 allele (*p* = 0.012, OR = 17.830, CI = 1.820–174.634), that lost significance after Bonferroni correction. Interestingly, an increase in the frequency of HLA-DRB1*01 allele was observed in mild disease patients compared to healthy controls (*p* = 0.002, OR = 2.927, CI = 1.537–5.577) which remained statistically significant even after Bonferroni correction (pc = 0.03).

## 4. Discussion

It is well known that HLA molecules play a critical role in viral protein presentation to T lymphocytes leading to an effective immune response and final clearance of the pathogen. Previous studies on HLA and SARS-CoV-1, the causative agent for a major infection outbreak in 2002–2003, generated mainly from Asian populations, reported conflicting results [5,6,7].

An increasing surge of reports on SARS-CoV-2 and HLA alleles has been observed since the pandemic outbreak. Nguyen et al. performed an in silico analysis of viral peptide-MHC class I binding affinity across HLA genotypes for all SARS-CoV-2 peptides. They suggested that HLA-A*02:02, -B*15:03, and -C*12:03 alleles have the greatest predicted capacity to present SARS-CoV-2 epitopes, whereas -A*25:01, -B*46:01, and -C*01:02 have the lowest, conferring disease protection and susceptibility, respectively [8]. Limited population studies on associations between HLA and SARS-CoV-2 infection which have identified a putative association of specific alleles with disease severity are also available. Novelli et al. reported a significant association of disease severity with HLA-DRB1*15:01, -DQB1*06:02, and -B*27:07 in a cohort of 99 Italian patients affected by a severe or extremely severe form of COVID-19 disease [9]. Poulton et al. analyzed data from 80 hospitalized COVID-19 patients, of mixed ethnicity from the UK, who were previously HLA typed to support transplantation, and also found a significant association of COVID-19 severity with the HLA-DQB1*06 allele [10]. Additionally, a study of HLA frequencies distributed in 82 Chinese patients, with mild or severe but not critical disease, revealed a significant increase in HLA-C*07:29 and -B*15:27 alleles in COVID-19 patients [11].

The present study assessed HLA frequencies in a cohort of Greek COVID-19 patients and identified a possible association of specific alleles with virus susceptibility and disease severity. Specifically, the HLA-DRB1*01 allele was shown to have an increased frequency in COVID-19 patients compared to healthy controls (Table 4) The specific allele was also found increased in both mild and hospitalized patients compared to healthy controls (Table 5) and also maintained significance following Bonferroni correction in the mild group suggesting that it may potentially act as a genetic predisposition factor for SARS-CoV-2 infection. An increased frequency of the HLA-DRB1*09 allele was also observed in hospitalized patients versus healthy controls (Table 5). Although DRB1* alleles were not reported in Nguyen et al. as potentially associated with severe COVID-19 based on binding affinity, the HLA-DRB1*08 allele has been associated with the highest risk for severe disease in a Sardinian population [12] as well as with mortality in an Italian cohort of patients [13] while Novelli et al. reported a significant association of disease severity with HLA-DRB1*15:01. Furthermore, alleles HLA-DRB1*01:01, HLA-DRB1*12:01, and HLA-DRB1*14:04 have also been associated with COVID-19 severity in a Chinese population of COVID-19 patients although the statistical significance was not maintained after Bonferroni correction [14]. Taken together, the above findings raise the question of whether alleles of the specific locus could play a possible role in the progression of COVID-19, however, as HLA frequencies are highly influenced by ethnicity, large multinational studies are needed in order to confirm such observations with the same patient criteria and group analysis.

Distinct patterns of allele frequencies distribution between COVID-19 patients with mild symptoms and severe and/or critical disease (hospitalized) were also identified. Specifically, an increase in -A*11, -A*23 alleles was observed in hospitalized patients compared to both healthy and mild and healthy respectively (Table 5) suggesting a possible association of the specific alleles with disease severity. An association of the HLA-A*11 with increased mortality in ICU COVID-19 patients has been previously reported [15] while the HLA-A*11:01 was associated with disease severity [14]. Furthermore, a decrease in the frequency of HLA-C*12 was found in the hospitalized group of patients compared to healthy controls (Table 5) suggesting a potentially protective role of the specific allele in disease severity. Interestingly, allele HLA-C*12 was also reported in an in silico analysis as a potential cross-protective allele [8]. A summary of the observations of studies related to the current study findings can be viewed in Table 6.

The main advantages of this study are the enrollment of an ethnically homogenous patient cohort of Greek origin only and the inclusion of hospitalized and non-hospitalized patients. However, the relatively small number of patients consists of a possible limitation and therefore further research on a larger cohort of patients is essential in order to confirm the specific findings.

## 5. Conclusions

The current study identified a potential association of the HLA-DRB1*01 allele with COVID-19 susceptibility and severity in a Greek cohort of COVID-19 patients. Although significant, our findings need further investigation in larger multicenter studies in order to clarify whether HLA genes play a role in the host susceptibility to SARS-CoV-2 infection and ultimately lead to improved clinical practice and patient management.

## Figures and Tables

**Table 1:** Presence and duration of symptoms in COVID-19-positive patients who did not require hospitalization (mild). (*n* = 62, due to non-retrieved information).

	Male	Female
Gender	31	31
Age	47 (20 69)	47 (21 69)
PCR diagnosis	28 (84.84%)	24 (80.00%)
Symptoms	26 (78.78%)	29 (96.66%)
Temperature		
No (>37 °C)	6 (18.18%)	8 (26.66%)
Low (37.1–37.9 °C)	10 (30.30%)	9 (30.00%)
High (<38.0 °C)	12 (36.36%)	13 (43.33%)
Duration of temperature (days)		
Moderate (37.1–37.9 °C)	6.9 (20.9%)	7.02 (23.4%)
High (<38.0 °C)	7.1 (21.51%)	7.1 (23.66%)
Fatigue		
No	14 (42.42%)	13 (43.33%)
Moderate	10 (30.30%)	10 (33.33%)
Severe	9 (32.14%)	7 (23.33%)
Headache		
No	15 (45.45%)	11 (36.66%)
Mild	10 (30.30%)	18 (60.00%)
Severe	8 (24.24%)	1 (3.33%) *
Cough		
No	20 (60.60%)	18 (60.00%)
Mild	8 (24.24%)	10 (33.33%)
Severe	5 (15.15%)	2 (6.67%)
Dyspnea		
No	19 (57.57%)	24 (80.00%)
Mild	9 (32.14%)	5 (16.66%)
Severe	5 (15.15%)	1 (3.33%)
Diarrhea	9 (32.14%)	11 (36.66%)
Loss of smell	12 (42.85%)	18 (60.00%)
Loss of taste	13 (46.22%)	21 (70.00%) *
CT scan or X-ray	6 (21.42%)	3 (10.00%)

Data are presented as the mean ± SD or absolute number (percentage of group). * *p* < 0.05 for comparisons between female and male.

**Table 2:** Demographic data and symptoms of COVID-19 patients who required hospitalization. (*n* = 45, due to non-retrieved information).

	Male	Female
Gender	33	12
Age	65.00 (29 85)	66.00 (38 86)
BMI	26.52	26.65
Smoking	8 (24, 24%)	1 (8.33%)
Comorbidities		
Asthma	1 (3.03%)	0 (0.00%)
Chronic obstructive pulmonary disease	0 (0.00%)	0 (0.00%)
Hypertension	14 (32.14%)	4 (23.33%)
Hyperlipidemia	5 (15.15%)	2 (16.66%)
Diabetes	6 (18.18%)	0 (0.00%)
Chronic artery disease	7 (21.21%)	0 (0.00%)
Chronic kidney disease	0 (0.00%)	0 (0.00%)
Days of illness prior to hospital/ICU admission	7.12 ± 2.87	5.83 ± 2.55
Symptoms at hospital/ICU admission		
Temperature (>37 °C)	29 (87.87%)	12 (100.00%)
Cough	17 (51.51%)	10 (83.33%)
Dyspnea	14 (42.42%)	6 (50.00%)
Fatigue	6 (18.18%)	3 (25.00%)
Diarrhea	1 (3.03%)	1 (8.33%)
Loss of smell	1 (3.03%)	1 (8.33%)
Loss of taste	1 (3.03%)	1 (8.33%)

**Table 3:** Clinical examination and laboratory parameters of COVID-19 patients who required hospitalization on admission to hospital or ICU.

	Male	Female	Normal Range
Chest X-ray Diffuse Infiltrates Infiltrates right Infiltrates left No infiltrates	33.00 (100.00%) 24.00 (72.72%) 2.00 (6.06%) 1.00 (3.03%) 2.00 (6.06%)	12.00 (100.00%) 12.00 (100.00%) - - -	
Computerized tomography (CT) Scan Ground glass	18.00 (54.54%)	6.00 (50.00%)	
PO_2_/FIO_2_	225.08 ± 88.34	218.59 ± 81.25	
Comorbidities			
Asthma	1.00 (3.03%)	0.00 (0.00%)	
Chronic obstructive pulmonary disease	0.00 (0.00%)	0.00 (0.00%)	
Hypertension	14.00 (43.75%)	4.00 (33.33%)	
Hyperlipidemia	5.00 (15.15%)	2.00 (16.66%)	
Diabetes	6.00 (18.18%)	0.00 (0.00%)	
Chronic artery disease	7.00 (21.21%)	0.00 (0.00%)	
Chronic kidney disease	0.00 (0.00%)	0.00 (0.00%)	
Laboratory (baseline)			
Neutrophils (%)	79.13 ± 12.05	79.21 ± 13.73	40–70
Lymphocytes (%)	15.22 ± 10.16	16.45 ± 11.25	25–45
Platelets	236091± 98854	221455 ± 78898	140–150
C-reactive protein	13.92 ± 12.06	14.80 ± 9.42	<0.5 mg/dL
Troponin	50.79 ± 112.2	23.82 ± 44.73	24–30 pg/mL
Urea	43.67 ± 26.00	34.91 ± 19.11	10–50 mg/dL
Creatinine	1.04 ± 0.34	0.80 ± 0.27 *	0.6–1.4 mg/dL
Aspartate aminotransferase	51.52 ± 44.10	46.91± 21.28	5–37 IU/L
Alanine transaminase	41.15 ± 28.17	44.73 ± 18.75	5–40 IU/L
Gamma-glutamyltransferase	70.67 ± 72.52	66.00 ± 59.24	7–49 IU/L
Lactate dehydrogenase	482.5 ± 294.6	402.6 ± 133.8	<225 IU/L
Albumin	3.54 ± 0.63	3.50 ± 0.41	3.5–5.0 g/dL
Duration of hospital/ICU stay (days)	23.84 ± 18.18	18.91± 14.42	
Outcome (survivors)	23.00 (63.63%)	10.00 (83.33%)	

Data are presented as the mean ± SD or absolute number (percentage of group). * *p* < 0.05 for comparisons between female and male. (male: *n* = 33 and female: *n* = 12).

**Table 4:** HLA allele frequencies (%) in COVID-19 patients and healthy controls. HLA allele frequency distribution in COVID-19 patients (*n* = 125) with a median age of 53 years (range 20–86) compared to a panel of age-matched healthy individuals (*n* = 165) with a median age of 53 years (range 41–75). *p* calculated by Fisher’s exact test and pc after applying Bonferroni correction.

HLA Allele	*F*%		*p*	pc
	Controls (n = 165)	COVID-19 (n = 125)		
DRB1*01	10.76	24.8	0.32	3.2
RB1*09	0.002	0.03	0.024	NS

**Table 5:** HLA allele frequencies in COVID-19 patients of Greek origin. Table showing frequencies of all alleles identified in different groups of COVID-19 patients in a Greek cohort as well as in a panel of healthy individuals of the same origin. The table shows HLA allele frequency distribution in mild (*n* = 69) with a median age of 46 years (range 20–69) and hospitalized COVID-19 patients (*n* = 56) with a median age of 62 years (range 29–86). A reference panel of healthy individuals (*n* = 316) with a median age of 44 years (range = 1–84) has been included for comparisons. *p* was calculated by Fisher’s exact test and pc after Bonferroni correction. * *p* < 0.05 vs. healthy, ^#^ *p* < 0.05 vs. hospitalized, ^$^ *p* < 0.05 vs. healthy, ^$$^ *p* < 0.05 vs. healthy. pc was non-significant for all comparisons apart from DRB1*01 allele between mild and healthy (pc = 0.03). *p* was calculated by Fisher’s exact test and pc after Bonferroni correction.

**Locus A**		***F*%**		**Locus B**		***F*%**		**Locus C**		***F*%**	
	**healthy**	**mild**	**hospitalized**		**healthy**	**mild**	**hospitalized**		**healthy**	**mild**	**hospitalized**
A*01	20.89	18.84	14.29	B*07	9.18	10.14	12.5	C*01	6.96	5.80	3.57
A*02	49.05	57.97	50.00	B*08	8.54	8.70	5.36	C*02	12.97	18.84	16.07
A*03	18.04	15.94	8.93	B*13	6.01	2.90	1.79	C*03	9.18	20.29	8.93
A*11	12.66	10.14	25.00 *^,#^	B*14:01 (B64)	0.63	0.00	0.00	C*04	28.48	15.94 ^#^	35.71
A*23	5.06	5.80	14.29 *	B*14:02 (B65)	3.48	7.25	5.36	C*05	5.70	5.80	3.57
A*24	22.78	24.64	28.57	B*15:01 (B62)	2.22	4.35	5.36	C*06	15.82	7.25	19.64
A*25	0.95	0.00	1.79	B*15:17 (B63)	0.63	1.45	1.79	C*07	44.3	39.13	39.29
A*26	11.71	7.25	7.14	B*15:10 (B71)	0.95	1.45	0.00	C*08	4.43	7.25	5.36
A*29	3.16	2.9	5.36	B*15:03 (B72)	0.63	1.45	0.00	C*12	31.01	33.33	16.07 *^,#^
A*30	6.01	4.35	5.36	B*18	27.85	17.39	23.21	C*14	7.28	8.70	10.71
A*31	4.11	2.90	5.36	B*27	4.43	4.35	3.57	C*15	14.24	11.59	14.29
A*32	11.08	18.84	7.14	B*35	30.06	20.29	37.5	C*16	5.06	7.25	7.14
A*33	3.16	4.35	7.14	B*37	1.90	1.45	5.36	C*17	4.11	5.80	3.57
A*66	1.27	2.90	1.79	B*38	5.38	2.90	3.57	C*18	0.00	0.00	1.79
A*68	8.54	14.49	5.36	B*39	6.65	13.04	3.57				
A*69	0.95	0.00	1.79	B*40:01 (B60)	1.58	5.80	0.00				
				B*40:02 (B61)	4.11	11.59 ^$^	3.57				
				B*41	4.75	7.25	5.36				
				B*44	11.08	18.84	16.07				
				B*45	0.32	0.00	0.00				
				B*47	1.27	0.00	1.79				
				B*49	4.75	5.80	5.36				
				B*50	2.22	0.00	3.57				
				B*51	27.85	24.64	30.36				
				B*52	6.33	5.80	1.79				
				B*53	0.63	0.00	0.00				
				B*55	4.11	8.70	5.36				
				B*56	0.95	0.00	0.00				
				B*57	3.16	1.45	8.93				
				B*58	2.85	1.45	1.79				
				B*73	1.27	2.90	0.00				
** Locus DR **		***F*** **%**		**Locus DQ**		***F*** **%**					
	**healthy**	**mild**	**hospitalized**		**healthy**	**mild**	**hospitalized**				
DRB1*01	10.76	26.09 ^$$^	23.21 *	DQB1*02	27.22	27.54	19.64				
DRB1*03:01 (DR17)	15.82	14.49	8.93	DQB1*03:01 (DQ7)	55.7	50.72	48.21				
DRB1*04	16.14	17.39	14.29	DQB1*03:02 (DQ8)	10.76	10.14	10.71				
DRB1*07	11.71	13.04	12.5	DQB1*03:03 (DQ9)	2.53	4.35	7.14				
DRB1*08	2.53	1.45	7.14	DQB1*04	3.16	1.45	3.57				
DRB1*09	0.32	1.45	5.36 *	DQB1*05	46.52	63.77	55.36				
DRB1*10	2.85	2.90	0.00	DQB1*06	22.47	23.19	32.14				
DRB1*11	50.63	43.48	39.29								
DRB1*12	2.22	1.45	3.57								
DRB1*13	16.77	20.29	25.00								
DRB1*14	10.44	10.14	10.71								
DRB1*15	11.39	10.14	14.29								
DRB1*16	22.47	30.43	26.79								

**Table 6:** Allele associations with COVID-19.

Allele	*p*	pc	Association	(Reference)
DRB1*15:01	0.0015	0.048	Disease Susceptibility	(9)
DRB1*08:01	0.002	0.028	Disease severity	(12)
DRB1*01:01	0.02	NA	Disease severity	(14)
DRB1*12:01	0.045	NA	Disease severity	(14)
DRB1*14:04	0.01	NA	Disease severity	(14)
A*11	0.04	NA	Mortality	(15)
A*11:01	0.008	NA	Disease severity	(14)

## Data Availability

The data that support the findings of this study are available on request from the corresponding author. The data are not publicly available due to privacy or ethical restrictions.

## Supplementary Materials

The following are available online at https://www.mdpi.com/article/10.3390/life11101017/s1, Table S1: HLA allele frequencies in COVID-19 patients of Greek origin. Table showing frequencies of all alleles identified in different groups of COVID-19 patients in a Greek cohort as well as in a panel of healthy individuals of the same origin. Table S2: HLA allele frequencies in COVID-19 patients of Greek origin who required hospitalization according to gender, Table S3: HLA allele frequencies in healthy individuals of Greek origin according to gender.
