# Supplementary material for: Demographic, Clinical and Immunogenetic Profiles of a Greek Cohort of COVID-19 Patients
Source: Life (Basel). 2021 Sep 27;11(10):1017. doi: 10.3390/life11101017 (PMC8541537; doi:10.3390/life11101017)
Supplement: Supplementary file 1 [file life-11-01017-s001.zip › life-1365323-supplementary.pdf]

Table S1. HLA allele frequencies in COVID-19 patients of Greek origin with mild symptoms according to gender.

**Locus A**	**F%**		**Locus B**	**F%**		**Locus C**	**F%**	
	**male**	**female**		**male**	**female**		**male**	**female**
**A*01**	21.05	16.13	B*07	13.16	11.11	C*01	2.63	9.68
**A*02**	63.16	51.61	B*08	5.26	12.9	C*02	13.16	25.81
**A*03**	21.05	9.68	B*13	5.26	0.00	C*03	18.42	22.58
**A*11**	7.89	12.9	B*14:01 (B64)	0.00	0.00	C*04	15.79	16.13
**A*23**	7.89	3.23	B*14:02 (B65)	7.89	6.45	C*05	10.53	0.00
**A*24**	13.16	38.71	B*15:01 (B62)	0.00	9.68	C*06	13.16	0.00
**A*25**	0.00	0.00	B*15:17 (B63)	0.00	3.23	C*07	36.84	41.94
**A*26**	5.26	9.68	B*15:10 (B71)	2.63	0.00	C*08	7.89	6.45
**A*29**	2.63	3.23	B*15:03 (B72)	0.00	3.23	C*12	42.11	22.58
**A*30**	5.26	3.23	B*18	13.16	22.58	C*14	10.53	6.45
**A*31**	2.63	3.23	B*27	2.63	6.45		10.53	12.9
**A*32**	23.68	12.9	B*35	21.05	19.35	C*15	2.63	12.9
**A*33**	5.26	3.23	B*37	2.63	0.00	C*16	5.26	6.45
**A*66**	2.63	3.23	B*38	5.26	0.00	C*17	0.00	0.00
**A*68**	10.53	19.35	B*39	15.79	9.68	C*18		
	0.00	0.00	B*40:01 (B60)	7.89	3.23			
			B*40:02 (B61)	13.16	9.68			
			B*41	7.89	6.45			
**A*69**			B*44	18.42	19.35			
			B*45	0.00	0.00			
			B*47	0.00	0.00			
			B*49	10.53	0.00			
			B*50	0.00	0.00			
			B*51	15.79	35.48			
			B*52	10.53	0.00			
			B*53	1.16	0.00			
			B*55	7.89	9.68			
			B*56	0.00	0.00			
			B*57	2.63	0.00			
			B*58	0.00	3.23			
			B*73	2.63	3.23			

**Locus DR**	**F%**		**Locus DQ**	**F%**	
	**male**	**female**		**male**	**female**
**DRB1*01**	28.95	22.57	DQB1*02	23.68	32.26
**DRB1*03:01 (DR17)**	5.26	25.81	DQB1*03:01 (DQ7)	50.00	51.61
**DRB1*04**	13.16	22.58	DQB1*03:02 (DQ8)	7.89	12.9
**DRB1*07**	0.00	3.23	DQB1*03:03 (DQ9)	7.89	0.00
**DRB1*08**	0.00	3.23	DQB1*04	0.00	3.23
**DRB1*09**	2.63	0.00	DQB1*05	68.42	58.06
**DRB1*10**	2.63	3.23	DQB1*06	26.32	12.9
**DRB1*11**	42.11	45.16			
**DRB1*12**	0.00	3.23			
**DRB1*13**	18.42	22.58			
**DRB1*14**	10.53	9.68			
**DRB1*15**	18.42	0.00			
**DRB1*16**	28.95	32.26			

Table S2. HLA allele frequencies in COVID-19 patients of Greek origin who required hospitalisation according to gender.

Locus A	F%		Locus B	F%		Locus C	F%	
	male	female		male	female		male	female
A*01	15.79	11.11	B*07	13.16	11.11	C*01	5.26	0.00
A*02	55.26	38.88	B*08	5.26	5.55	C*02	15.79	16.66
A*03	7.89	11.11	B*13	2.63	1.32	C*03	7.89	11.11
A*11	18.42	38.88	B*14:01 (B64)	0.00	0.00	C*04	34.21	38.88
A*23	13.16	16.66	B*14:02 (B65)	5.26	5.55	C*05	5.26	0.00
A*24	31.58	22.22	B*15:01 (B62)	7.89	0.00	C*06	15.79	27.77
A*25	0.00	5.55	B*15:17 (B63)	0.00	5.55	C*07	39.47	38.88
A*26	7.89	5.55	B*15:10 (B71)	0.00	0.00	C*08	5.26	5.55
A*29	7.89	0.00	B*15:03 (B72)	0.00	0.00	C*12	15.79	16.66
A*30	5.26	5.55	B*18	28.95	11.11	C*14	13.16	5.55
A*31	5.26	5.55	B*27	2.63	5.55	C*15	13.16	16.66
A*32	7.89	5.55	B*35	34.21	44.44	C*16	10.53	0.00
A*33	5.26	11,11	B*37	5.26	5.55	C*17	5.26	0.00
A*66	2.63	0.00	B*38	5.26	0.00	C*18	0.00	0.00
A*68	2.63	11.11	B*39	0.00	11.11			
A*69	0.00	5.55	B*40:01 (B60)	0.00	0.00			
			B*40:02 (B61)	2.63	5.55			
			B*41	5.26	5.55			
			B*44	15.79	16.66			
			B*45	0.00	0.00			
			B*47	0.00	5.56			
			B*49	2.63	11.11			
			B*50	5.26	0.00			
			B*51	36.84	16.66			
			B*52	2.63	0.00			
			B*53	0.00	0.00			
			B*55	2.63	11.11			
			B*56	0.00	0.00			
			B*57	5.26	16.66			
			B*58	2.63	0.00			
			B*73	0.00	0.00			

**Locus DR**	**F%**		**Locus DQ**	**F%**	
	**male**	**female**		**male**	**female**
DRB1*01	21.05	27.77	DQB1*02	15.79	27.77
DRB1*03:01 (DR17)	5.26	16.66	DQB1*03:01 (DQ7)	55.26	33.33
DRB1*04	10.53	22.22	DQB1*03:02 (DQ8)	5.26	22.22
DRB1*07	10.53	16.66	DQB1*03:03 (DQ9)	5.26	33.33
DRB1*08	10.53	0.00	DQB1*04	5.26	0.00
DRB1*09	5.26	5.55	DQB1*05	52.63	61.11
DRB1*10	0.00	0.00	DQB1*06	36.84	22.22
DRB1*11	44.74	27.77			
DRB1*12	2.63	5.55			
DRB1*13	31.58	11.11			
DRB1*14	13.16	5.55			
DRB1*15	13.16	16.66			
DRB1*16	23.68	33.33			

Table S3. HLA allele frequencies in healthy individuals of Greek origin according to gender.

Locus A	F%		Locus B	F%		Locus C	F%	
	male	female		male	female		male	female
A*01	25.00	15.97	B*07	7.56	11.11	C*01	6.40	7.64
A*02	45.35	53.47	B*08	11.63	4.86	C*02	11.63	14.58
A*03	18.6	17.36	B*13	7.56	4.17	C*03	5.81	13.19
A*11	13.37	11.81	B*14:01 (B64)	0.58	0.69	C*04	31.4	25.00
A*23	4.65	5.56	B*14:02 (B65)	2.33	4.86	C*05	6.40	4.86
A*24	21.51	24.31	B*15:01 (B62)	1.16	3.47	C*06	18.6	12.5
A*25	1.74	0.00	B*15:17 (B63)	0.58	0.69	C*07	41.86	47.22
A*26	12.79	10.42	B*15:10 (B71)	0.58	1.39	C*08	2.91	6.25
A*29	3.49	2.78	B*15:03 (B72)	0.58	0.69	C*12	31.98	29.86
A*30	5.81	6.25	B*18	25.00	31.25	C*14	8.72	5.56
A*31	3.49	4.86	B*27	3.49	5.56	C*15	13.95	14.58
A*32	11.05	11.11	B*35	32.56	27.08	C*16	6.4	3.47
A*33	5.26	3.47	B*37	1.74	2.08	C*17	5.23	2.78
A*66	1.74	0.69	B*38	5.81	4.86	C*18	0.00	0.00
A*68	8.72	8.33	B*39	6.98	6.25			
A*69	1.74	0.00	B*40:01 (B60)	1.16	2.08			
			B*40:02 (B61)	4.65	3.47			
			B*41	5.23	4.17			
			B*44	13.37	8.33			
			B*45	0.58	0.00			
			B*47	1.74	0.69			
			B*49	4.65	4.86			
			B*50	2.33	2.08			
			B*51	29.07	26.39			
			B*52	6.98	5.56			
			B*53	1.16	0.00			
			B*55	2.91	5.56			
			B*56	1.74	0.00			
			B*57	2.91	3.47			
			B*58	1.74	4.17			
			B*73	0.00	2.78			

**Locus DR**	**F%**		**Locus DQ**	**F%**	
	**male**	**female**		**male**	**female**
DRB1*01	10.47	11.11	DQB1*02	31.4	22.22
DRB1*03:01 (DR17)	17.44	13.89	DQB1*03:01 (DQ7)	58.14	52.78
DRB1*04	15.7	16.67	DQB1*03:02 (DQ8)	10.47	11.11
DRB1*07	13.37	9.72	DQB1*03:03 (DQ9)	1.74	3.47
DRB1*08	2.91	2.08	DQB1*04	4.07	2.08
DRB1*09	0.58	0.00	DQB1*05	40.7	53.47
DRB1*10	2.33	3.47	DQB1*06	22.67	22.22
DRB1*11	52.91	47.92			
DRB1*12	1.74	2.78			
DRB1*13	17.44	15.97			
DRB1*14	8.72	12.5			
DRB1*15	12.21	10.42			
DRB1*16	19.19	26.39			
